# Supplementary material for: Complexin inhibits spontaneous release and synchronizes Ca2+-triggered synaptic vesicle fusion by distinct mechanisms
Source: eLife. 2014 Aug 13;3:e03756. doi: 10.7554/eLife.03756 (PMC4130161; doi:10.7554/eLife.03756)
Supplement: Figure 2—source data 1. — The histograms (1 s time bin) are combinations of all respective repeat experiments, and normalized with respect to the number of associated SV vesicles. Exponential decay functions were fit to the histograms. The mock injection is an injection of Cy5 dye buffer without Ca2+; as expected, no Ca2+-triggered fusion was observed, that is, the mechanical disturbance of the injection does not cause fusion events. The table shows the number of spontaneous and Ca2+-triggered fusion events, the total number of analyzed traces (i.e., the number of associated vesicles), and the number of repeat experiments (N). DOI: http://dx.doi.org/10.7554/eLife.03756.003 [file elife03756s001.pptx]

## Slide 1
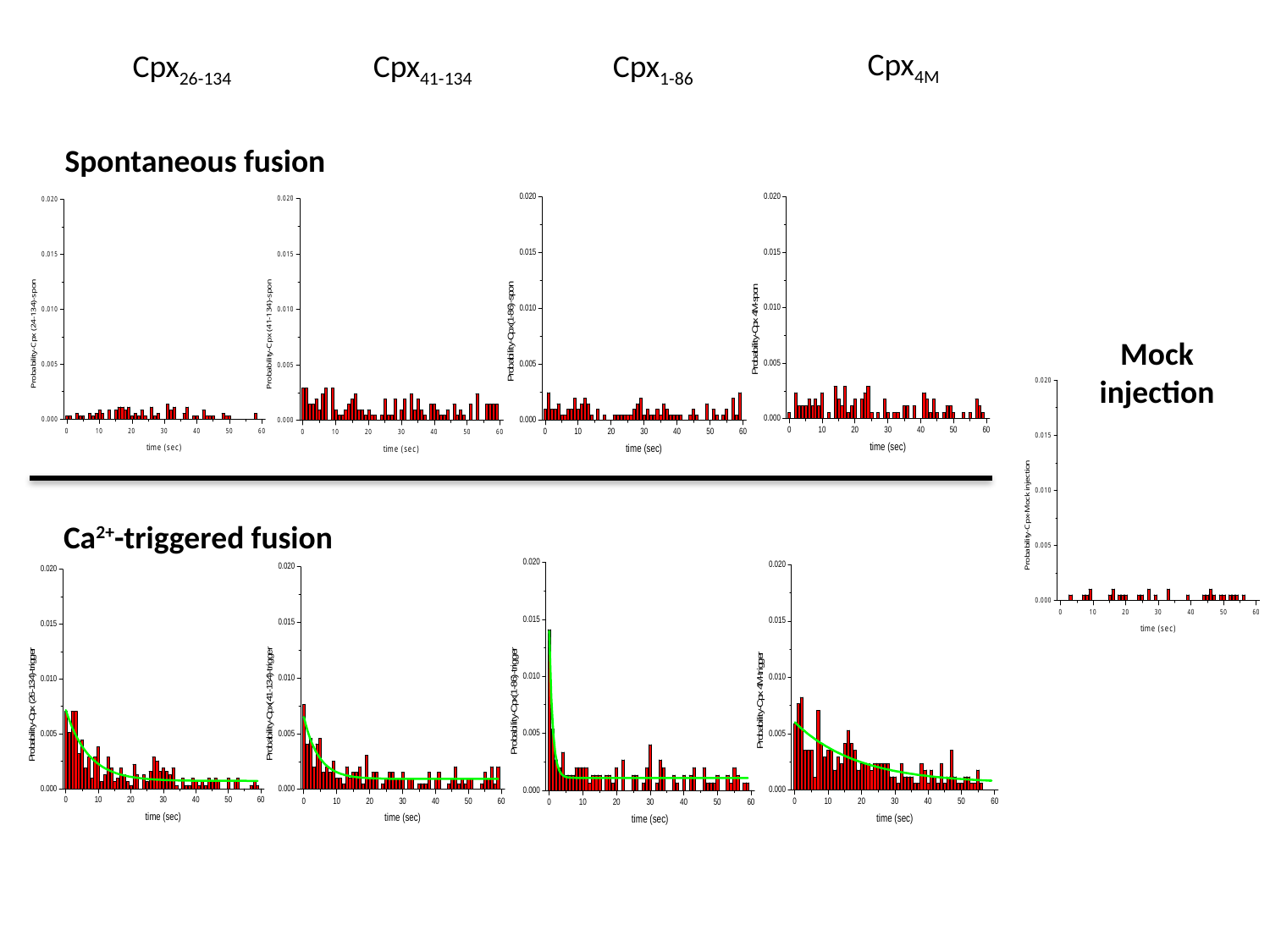

Cpx4M
Cpx26-134
Cpx41-134
Cpx1-86
Spontaneous fusion
Mock injection
Ca2+-triggered fusion

## Slide 2
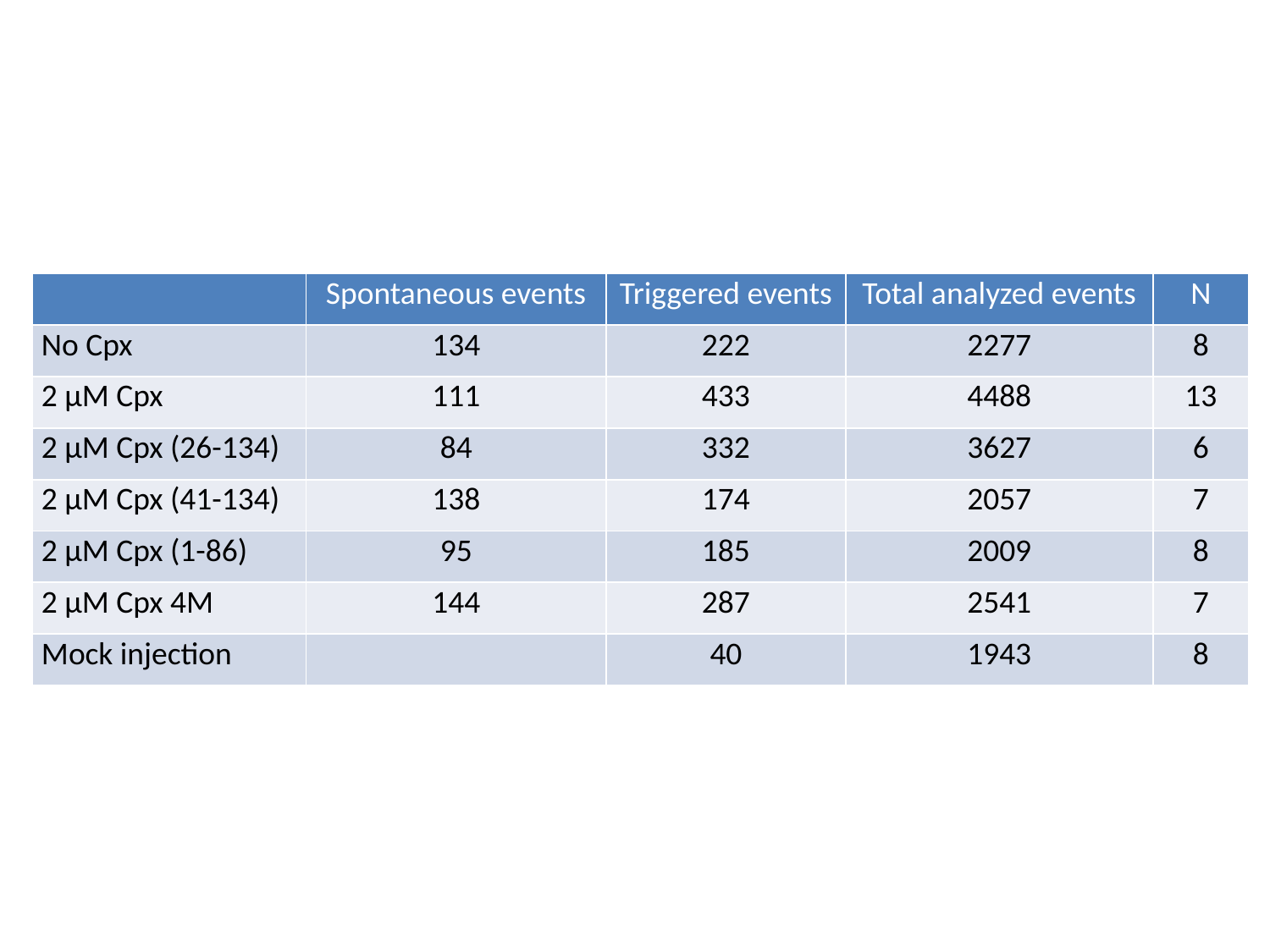

| | Spontaneous events | Triggered events | Total analyzed events | N |
| --- | --- | --- | --- | --- |
| No Cpx | 134 | 222 | 2277 | 8 |
| 2 µM Cpx | 111 | 433 | 4488 | 13 |
| 2 µM Cpx (26-134) | 84 | 332 | 3627 | 6 |
| 2 µM Cpx (41-134) | 138 | 174 | 2057 | 7 |
| 2 µM Cpx (1-86) | 95 | 185 | 2009 | 8 |
| 2 µM Cpx 4M | 144 | 287 | 2541 | 7 |
| Mock injection | | 40 | 1943 | 8 |
