# Supplementary material for: Complexin inhibits spontaneous release and synchronizes Ca2+-triggered synaptic vesicle fusion by distinct mechanisms
Source: eLife. 2014 Aug 13;3:e03756. doi: 10.7554/eLife.03756 (PMC4130161; doi:10.7554/eLife.03756)
Supplement: Figure 3—source data 1. — The histograms (1 s time bin) are combinations of all respective repeat experiments, and normalized with respect to the number of associated SV vesicles. Exponential decay functions were fit to the histograms. The table shows the number of spontaneous and triggered fusion events, the total number of analyzed traces, and the number of repeat experiments (N). DOI: http://dx.doi.org/10.7554/eLife.03756.006 [file elife03756s002.pptx]

## Slide 1
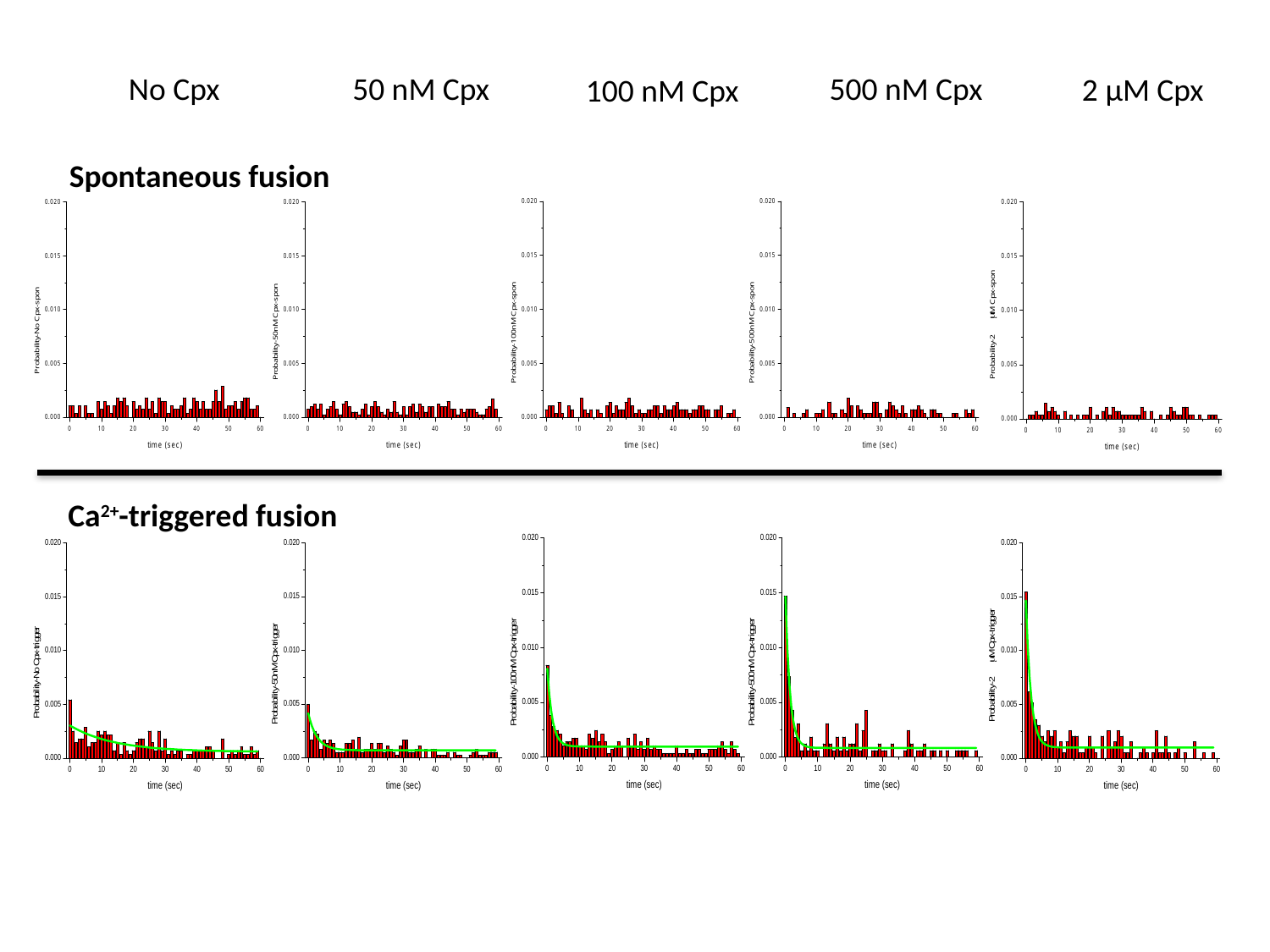

No Cpx
50 nM Cpx
500 nM Cpx
2 μM Cpx
100 nM Cpx
Spontaneous fusion
Ca2+-triggered fusion

## Slide 2
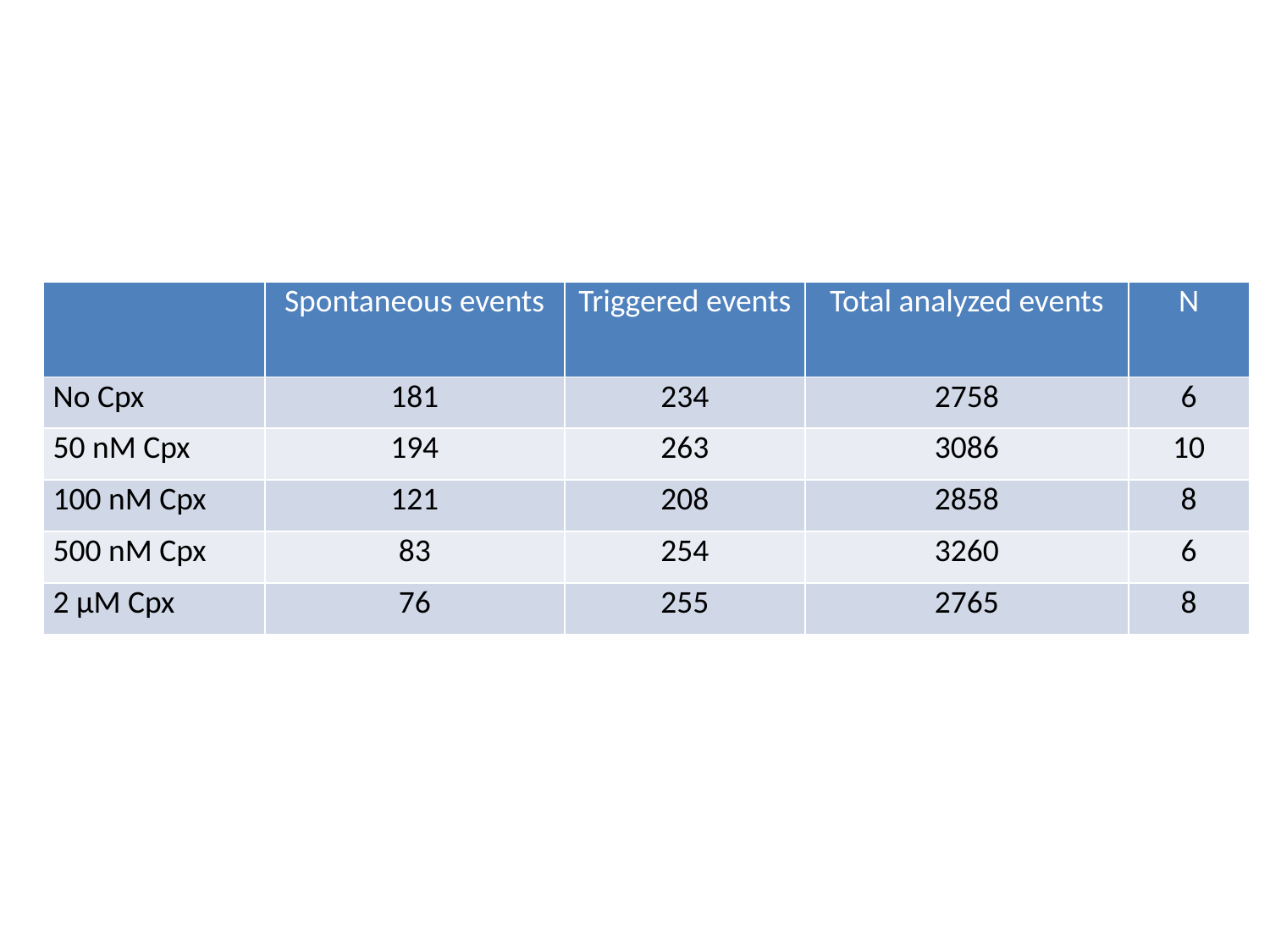

| | Spontaneous events | Triggered events | Total analyzed events | N |
| --- | --- | --- | --- | --- |
| No Cpx | 181 | 234 | 2758 | 6 |
| 50 nM Cpx | 194 | 263 | 3086 | 10 |
| 100 nM Cpx | 121 | 208 | 2858 | 8 |
| 500 nM Cpx | 83 | 254 | 3260 | 6 |
| 2 µM Cpx | 76 | 255 | 2765 | 8 |
