# Supplementary material for: Complexin inhibits spontaneous release and synchronizes Ca2+-triggered synaptic vesicle fusion by distinct mechanisms
Source: eLife. 2014 Aug 13;3:e03756. doi: 10.7554/eLife.03756 (PMC4130161; doi:10.7554/eLife.03756)
Supplement: Figure 4—source data 1. — The histograms (1 s time bin) are combinations of all respective repeat experiments, and normalized with respect to the number of associated SV vesicles. The table shows the number of spontaneous fusion events, the total number of analyzed traces, and the number of repeat experiments (N). DOI: http://dx.doi.org/10.7554/eLife.03756.008 [file elife03756s003.pptx]

## Slide 1
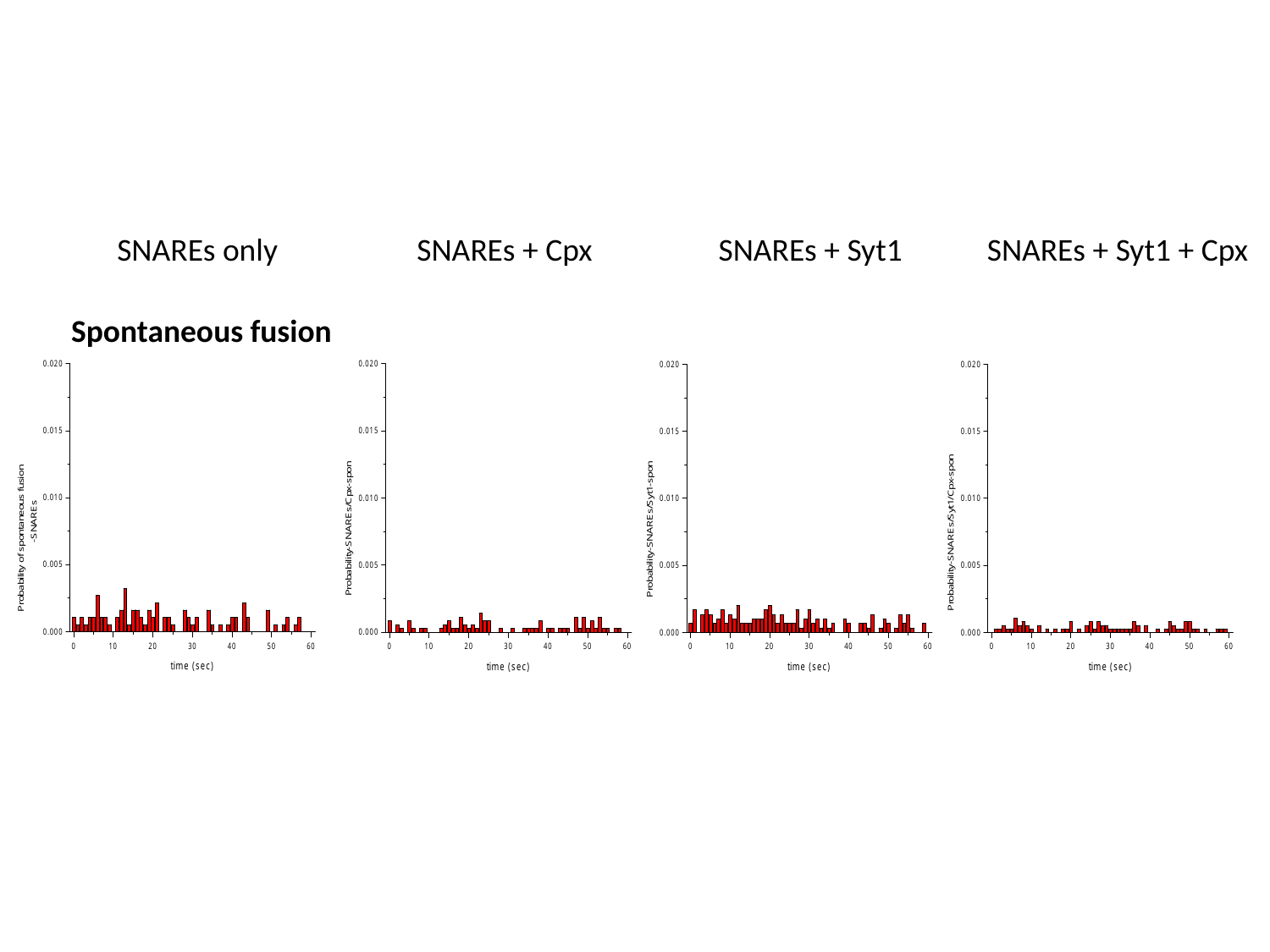

SNAREs + Syt1 + Cpx
SNAREs only
SNAREs + Cpx
SNAREs + Syt1
Spontaneous fusion

## Slide 2
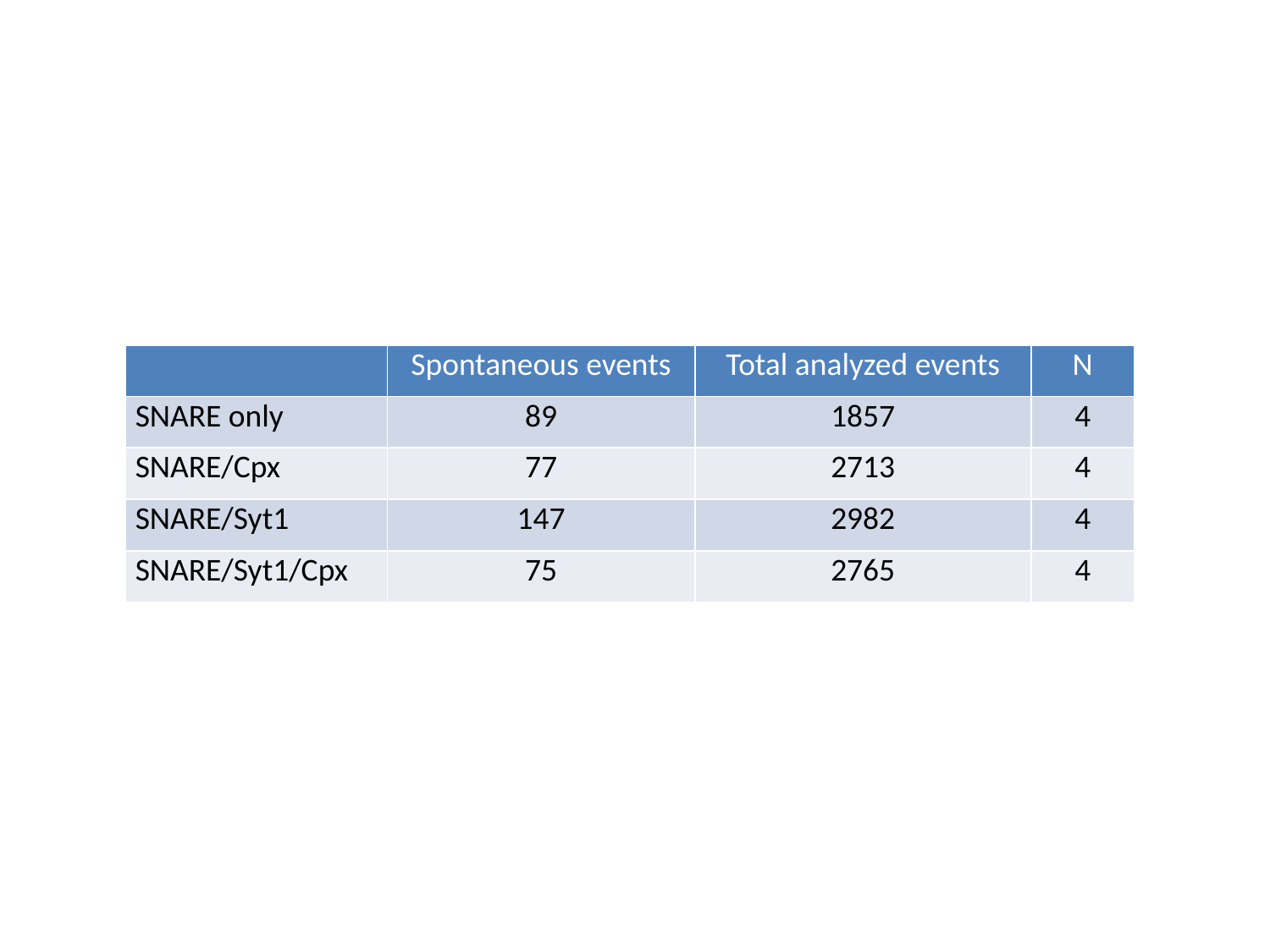

| | Spontaneous events | Total analyzed events | N |
| --- | --- | --- | --- |
| SNARE only | 89 | 1857 | 4 |
| SNARE/Cpx | 77 | 2713 | 4 |
| SNARE/Syt1 | 147 | 2982 | 4 |
| SNARE/Syt1/Cpx | 75 | 2765 | 4 |
